# Assessing the Spatiotemporal Spread Pattern of the COVID-19 Pandemic in Malaysia

**DOI:** 10.3389/fpubh.2022.836358

**Published:** 2022-03-04

**Authors:** Yoon Ling Cheong, Sumarni Mohd Ghazali, Mohd Khairuddin bin Che Ibrahim, Chee Cheong Kee, Nuur Hafizah Md Iderus, Qistina binti Ruslan, Balvinder Singh Gill, Florence Chi Hiong Lee, Kuang Hock Lim

**Affiliations:** ^1^Institute for Medical Research, National Institutes of Health, Ministry of Health Malaysia, Kuala Lumpur, Malaysia; ^2^Sector for Biostatistics and Data Repository, National Institutes of Health, Ministry of Health Malaysia, Shah Alam, Malaysia

**Keywords:** spatial autocorrelation, SaTScan, Moran's I, COVID-19, LISA, space-time scan

## Abstract

**Introduction:**

The unprecedented COVID-19 pandemic has greatly affected human health and socioeconomic backgrounds. This study examined the spatiotemporal spread pattern of the COVID-19 pandemic in Malaysia from the index case to 291,774 cases in 13 months, emphasizing on the spatial autocorrelation of the high-risk cluster events and the spatial scan clustering pattern of transmission.

**Methodology:**

We obtained the confirmed cases and deaths of COVID-19 in Malaysia from the official GitHub repository of Malaysia's Ministry of Health from January 25, 2020 to February 24, 2021, 1 day before the national vaccination program was initiated. All analyses were based on the daily cumulated cases, which are derived from the sum of retrospective 7 days and the current day for smoothing purposes. We examined the daily global, local spatial autocorrelation and scan statistics of COVID-19 cases at district level using Moran's I and SaTScan™.

**Results:**

At the initial stage of the outbreak, Moran's I index > 0.5 (*p* < 0.05) was observed. Local Moran's I depicted the high-high cluster risk expanded from west to east of Malaysia. The cases surged exponentially after September 2020, with the high-high cluster in Sabah, from Kinabatangan on September 1 (cumulative cases = 9,354; Moran's I = 0.34; *p* < 0.05), to 11 districts on October 19 (cumulative cases = 21,363, Moran's *I* = 0.52, *p* < 0.05). The most likely cluster identified from space-time scanning was centered in Jasin, Melaka (RR = 11.93; *p* < 0.001) which encompassed 36 districts with a radius of 178.8 km, from November 24, 2020 to February 24, 2021, followed by the Sabah cluster.

**Discussion and Conclusion:**

Both analyses complemented each other in depicting underlying spatiotemporal clustering risk, giving detailed space-time spread information at district level. This daily analysis could be valuable insight into real-time reporting of transmission intensity, and alert for the public to avoid visiting the high-risk areas during the pandemic. The spatiotemporal transmission risk pattern could be used to monitor the spread of the pandemic.

## Introduction

Coronavirus disease 2019 (COVID-19) which is caused by the severe acute respiratory syndrome coronavirus 2 (SARS-CoV-2), was first detected in Wuhan, China in December 2019. Until 4 November 2021, the pandemic COVID-19 has surpassed 248 million cases and 5 million deaths worldwide (1) with an estimated reproduction number or R_0_ value of 1.70 (*SD* = 0.57) (2). The total deaths due to COVID-19 have surpassed those of the Middle East respiratory syndrome (MERS) and severe acute respiratory syndrome (SARS), and the majority of the dead were elderly with history of comorbidities such as hypertension, diabetic, obese, and heart disease (3). Many countries implemented non-pharmaceutical interventions that include contact tracing, quarantine and isolation, universal lockdowns, closure of borders, schools and workplaces, physical distancing, and mask-wearing mandate, with varying effects due to different levels of compliances (4, 5). Although COVID-19 vaccination programs have been initiated since the end of December 2020 (6), the rapid emergence of SARS-CoV-2 variants of concern increased the complexity of controlling the disease (7, 8).

As of October 15, 2021, Malaysia had recorded 2,377,033 COVID-19 cases and 27,770 deaths and had the highest case fatality rate (as of September 2021) in Southeast Asia with 80.3 deaths per 100,000 cases (9). Malaysia experienced its first wave of COVID-19 cases from January 25 to February 16, 2020, with only 22 imported and local cases (10, 11). The second wave of the pandemic from February 27 till end of August resulted 9,340 confirmed cases and 127 deaths (12), which were mainly due to a religious mass gathering of an estimated 14,500 local and 1,500 overseas attendees in Sri Petaling, Selangor from February 27 to March 3, 2020 (10, 13, 14). A nationwide movement control order (MCO), which was a partial lockdown, was enforced from March 18, 2020, and subsequent conditional (CMCO) and the recovery MCOs (RMCO) successfully reduced cases and deaths (12). Malaysia experienced zero cases during the period of July 1, 2020 (15).

Studies on the spatiotemporal spread of diseases measure the diffusion and density of disease transmission. Identification of the spatiotemporal pattern and ability to predict the spread enables policymakers to plan mitigation strategies. A spatiotemporal study in China tracked the spread of COVID-19 from Wuhan city, to the Grand Bay Area to the east (16). In Brazil, COVID-19 was first detected in São Paulo with subsequent spread to the north of Brazil, estimation of deaths clustered 1 month before cases and took 17.3 and 32.3 days to reach 50 cases and deaths, respectively (17). A spatial extension of clusters ranging from 0.02 to 2 square kilometer and temporal durations of 6–13 days was identified along with 13 significant emerging clusters in Kuwait (18). In India, the COVID-19 case clustering tendency in 60 districts of western part of the country was observed using hotspot analysis (19).

In Malaysia, many studies have been done that described the characteristics and trend of the COVID-19 epidemic (20), which evaluates the effectiveness of the movement control orders (21), and response and other countermeasures (13, 14). However, studies on spatiotemporal distribution of COVID-19 in Malaysia are scarce. The few studies available were limited to analysis of spatiotemporal pattern of cases using monthly (22) or biweekly data at district level (23). Other studies examined state-level variations in cases and their interactions with air pollutant concentrations (24), or described epidemiological indicators by subregion (25). Spatiotemporal pattern analysis of disease transmission is vital in measuring the spatial dynamics of the epidemic for monitoring its occurrence, intensity, and direction of transmissibility. In this study, we investigated the spatiotemporal clustering pattern of COVID-19 cases in Malaysia specifically the district-level daily spatial autocorrelation of COVID-19 cases and identified spatiotemporal clusters of COVID-19 in Malaysia. The findings of this study could be used as a reference in preparation for similar outbreaks in the future.

## Materials and Methods

### Study Area and Period

Malaysia is the 6th highest populated country in Southeast Asia with an estimated population of 32.37 million in 2020 (26). Geographically, it is situated adjacent to the equator and consists of two major landmasses, Peninsular Malaysia to the west and Sabah and Sarawak (in Malaysian Borneo) to the east of the South China Sea. We included the study period from January 25, 2020 to February 24, 2021, 1 day before national vaccination program was initiated.

### Data Collection

This study was a retrospective cross-sectional study using district-level COVID-19 cases data in Malaysia. There is a total of 155 districts throughout the country. The base map of year 2019 and mid-year population data were obtained from the Department of Survey and Mapping Malaysia and the Department of Statistics Malaysia, respectively. The map and population were prepared based on the latest list of districts in the Malaysian COVID-19 open data GitHub repository (27). The base map was modified to accommodate 13 newly redelineated districts (Pokok Sena, Bagan Datuk, Kalabakan, Telupid, Beluru, Bukit Mabong, Kabong, Pusa, Sebauh, Tebedu, Telang Usan, Subis, and Tanjung Manis) using QGIS software v3.8. Data on confirmed COVID-19 cases and mortality were obtained from the same GitHub repository (28, 29). The definition of confirmed COVID-19 cases in this study is cases that were tested positive by real-time polymerase chain reaction (RT-PCR) (28). We analyzed the data at the district level. The federal territories of Putrajaya, Kuala Lumpur, and Labuan were treated as three distinct districts. We used current day and retrospective 7-day cumulative cases as the daily reported cases to account for the median incubation period of COVID-19 (30).

### The Spatial Autocorrelation Model

Global Moran's I spatial autocorrelation was utilized to determine whether COVID-19 cases were randomly distributed or clustered daily (31). The Moran's I index ranges from −1 to +1, where positive and negative values indicate positive and negative spatial autocorrelation, respectively, and 0 indicates spatial randomness. Neighbor weighting based on first-order queen contiguity was applied, which considers districts sharing common border as neighbors. Standardization of the weighting for common border is not applied, as this would introduce bias information to the districts that have more (or less) of common borders due to the size and shape of their district borders as compared to the rest. Langkawi and Labuan were excluded from the analysis as they are islands with tight border controls and require different measures of weight matrix.

The Local Indicator of Spatial Association (LISA) was also examined to identify the daily local spatial association of COVID-19 cases (32). Spatial association of neighboring districts was categorized as positively correlated with similar values (high-high, low-low) or negatively correlated with dissimilar value (high-low, low-high). Statistical significance of the spatial associations was tested using randomization based on 999 permutations (*p* < 0.05). We used the package “spdep” to calculate daily Global Moran's I in R software version 4.0.2, and the package “pygeoda” to obtain Local Moran's I statistics in Python.

### The Space-Time Scan Analysis

In addition to Moran's statistics, spatiotemporal heterogeneity of COVID-19 cases was also assessed using space-time scan statistics in SaTScan^TM^ v9.6 (33). The space-time scan statistic utilizes a cylindrical scanning window which has a circular geographical base and whose height corresponds to time (33). The base of the cylindrical window is centered at the centroids of the districts, whereas the height of window varies according to the user-defined study period. During a scan, the window is moved through space and time, which generates numerous cylinders varying in size and height, each reflecting a possible cluster. Then, probability models are used to determine the likelihood of finding cases within the window over the probability of finding cases outside it. The likelihood function for each window is calculated, and the size of the window restricted to a maximum of 50% of the population at risk to determine the maximum spatiotemporal cluster throughout the study period. In this study, the Poisson probability model was applied. The significance of each window–cluster was obtained through 999 iterations using Monte Carlo simulation. The temporal cluster size was set to “day.” No geographical overlap was used as criteria for reporting secondary clusters. The datasets were prepared in three files: the confirmed case file, the population by district, and the geographic coordinates for the centroids of each district.

**Figure 1:**
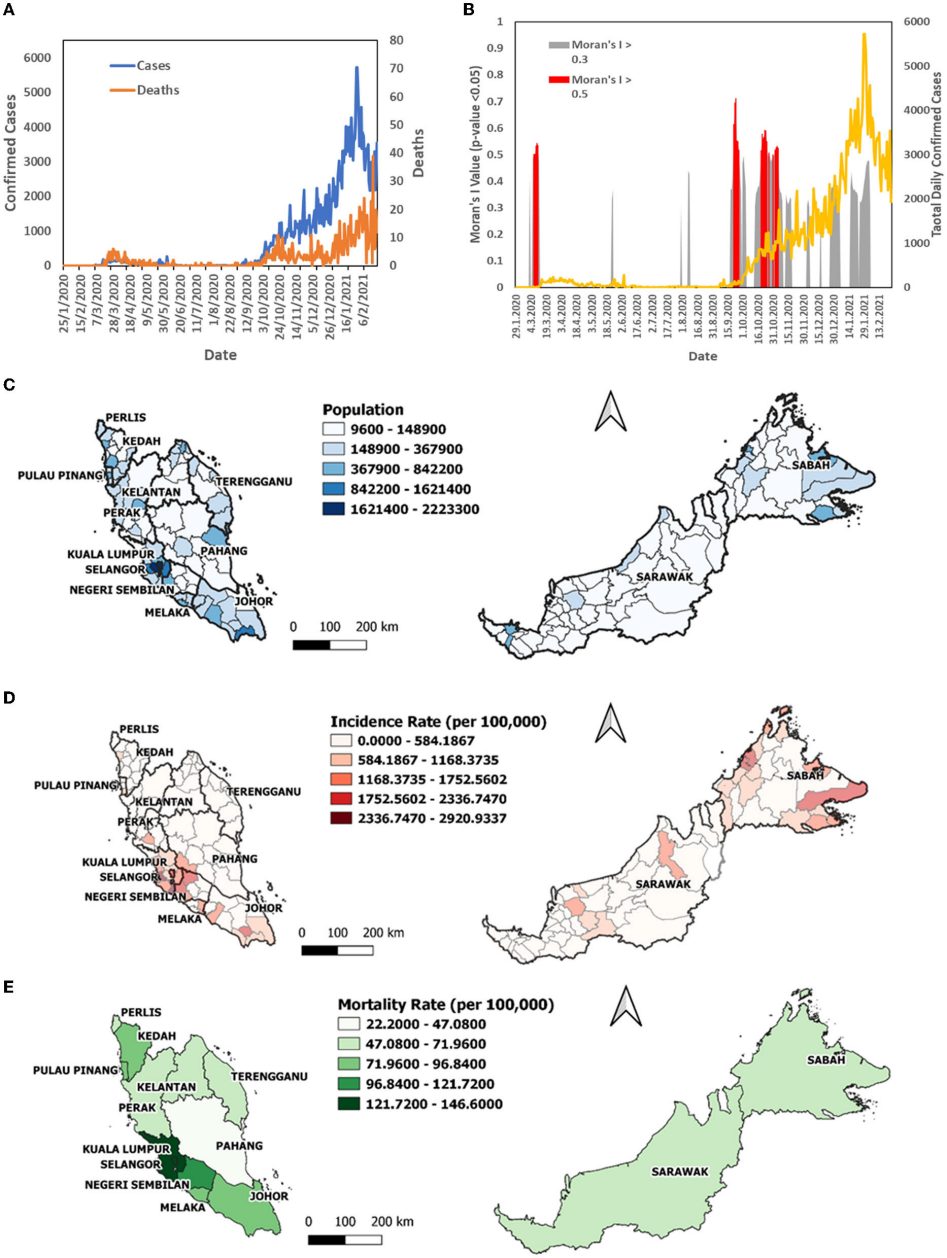
The COVID-19 case profile in Malaysia from January 25, 2020 to February 24, 2021; **(A)** total daily confirmed cases, daily mortality; **(B)** Moran's *I* >0.5 over the same period; **(C)** map of population by district; **(D)** map of incidence rate per 1,00,000 population by district; **(E)** map of mortality rate per 1,00,000 population by state.

Throughout the study period, high peak of cases occurred after the end of September 2020 (Figure 1A). Hence, we divided the study period into two, (1) period of first case until February 24, 2021 and (2) period of first case until 1 day before earliest cluster date in period 1. The second period is to identify the smaller clustering events before the surge of cases after September 2020.

## Results

A total of 291,774 confirmed COVID-19 cases and 1,093 COVID-19 deaths were reported from January 25, 2020 to February 24, 2021. Total daily cases and total daily mortality (Figure 1A) increased sharply after September 2020 initiating the third wave of the pandemic in Malaysia. The daily Moran's I value fluctuated across this time period, with positive spatial autocorrelation above 0.3 (*p* < 0.05, permutation of 999) (Figure 1B). The higher Moran's I value above 0.5 (*p* < 0.05, permutation of 999) was reported in March, September, October, and November (Figure 1B; in red), which were also the initial period of second and third waves. The highest incidence rate over the period was observed in Sepang (2,920.93 cases per 100,000) and Klang (2,668.72) in Selangor, the state with highest population (Figure 1C), followed by Putatan in Sabah (2,325.58), Jelebu in Negeri Sembilan (2,212.25), and Kulai (2,189.96) in Johor. Sabah districts constituted half of the top ten districts with the highest incidence rate (Figure 1D). The states with the highest mortality rate were Selangor (146.59 COVID-19 deaths per 100,000), Labuan (145.49), Kuala Lumpur (137.65), and Negeri Sembilan (110.86) (Figure 1E).

**Figure 2:**
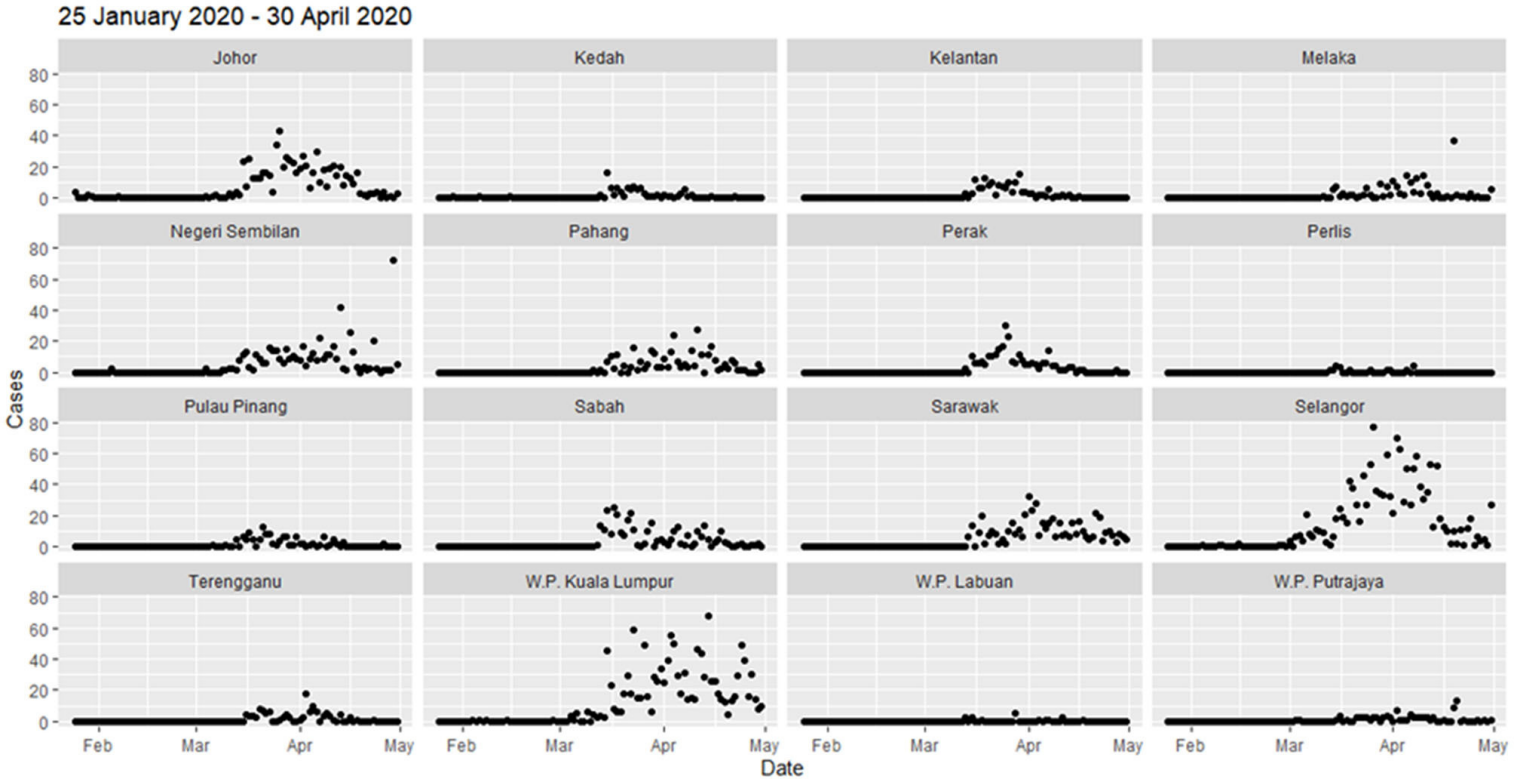
Total COVID-19 new cases by state from January 25, 2020 to April 30, 2020.

**Figure 3:**
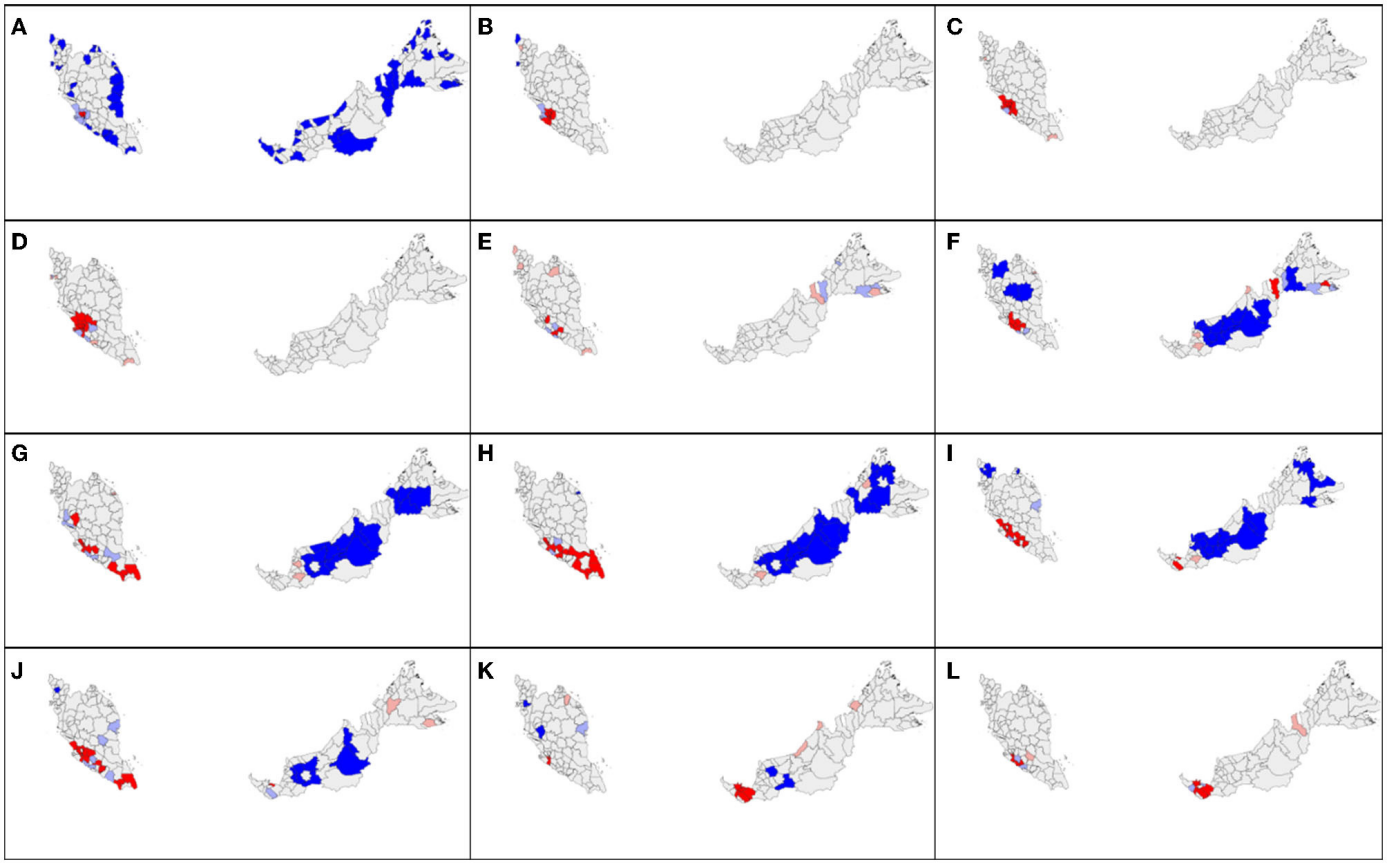
Spatial autocorrelation distribution of COVID-19 new cases by district in Malaysia at cumulative sum of current and 7 days retrospectively in 2020 on **(A)** February 4; **(B)** March 3; **(C)** March 6; **(D)** March 11; **(E)** March 14; **(F)** March 17; **(G)** March 26; **(H)** March 30; **(I)** April 12; **(J)** April 15; **(K)** April 21; **(L)** April 30. Red color indicates high-high cluster, pink indicates high-low, blue indicates low-low, and light blue indicates low-high cluster.

The daily district-level local spatial autocorrelation analysis showed the dispersal of high-risk clusters from small area to larger extent. In the initial period of 4 months from January to April 2020, the COVID-19 cases were reported in all states, but the highest in Selangor (1,432 cases), Kuala Lumpur (1,232), and Johor (663) (Figure 2). Most cases were reported after middle of March 2020. Since the COVID-19 cases first detected on January 25, 2020, the initial high-high cluster was observed in Petaling in Selangor and Kuala Lumpur on February 4 (Figure 3). The clusters expanded to four surrounding districts in Selangor (Hulu Langat, Gombak, Sepang, and Kuala Langat) and Putrajaya on March 3, 2020, subsequently further included the neighboring state, Seremban in Negeri Sembilan on March 6, 2020 and Bentong in Pahang on March 11, 2020 (Moran's I: 0.54, *p* < 0.05) (Figure 3). On March 14, the high-high cluster in Selangor was reduced to only one district (Hulu Langat), but started to move south (Tampin, Negeri Sembilan; Alor Gajah, Melaka). Within 2 weeks, the high-high cluster had covered a larger area of nine districts in several states in the south of Peninsula Malaysia (Kota Tinggi, Batu Pahat, Pontian and Kulai, Johor; Seremban and Kuala Pilah, Negeri Sembilan; Batang Padang, Perak; Petaling and Sepang, Selangor) (Figure 3). Four days later, the high-high cluster included 12 districts with the addition of three districts (Mersing and Segamat, Johor; Kuala Pilah, Negeri Sembilan). From the middle to end of April, Sarawak had a high-high cluster in two districts (Asajaya and Serian), which subsequently expanded to three adjacent districts (Samarahan, Simunjan and Kuching), whereas Selangor experienced a reduction in cases (Figure 3).

In the subsequent 3 months from May to July 2020, the total number of cases remained below 100 for all states except Kuala Lumpur (1,245 cases), Selangor (690), Negeri Sembilan (516), and Sarawak (171) with minor fluctuation (Figure 2 and Supplementary Figure S1). On May 23, high-high cluster was reported in more neighboring districts of Selangor including Bentong in Pahang and Seremban in Negeri Sembilan, with total of 9 districts (Supplementary Figure S2). The number of districts in the high-high cluster reduced to six and was situated only in Negeri Sembilan and Melaka on 16 June. Till the end of July (July 29), west Sarawak (Samarahan, Lundu, Kuching, Serian) and Perlis showed a high-high cluster.

**Figure 4:**
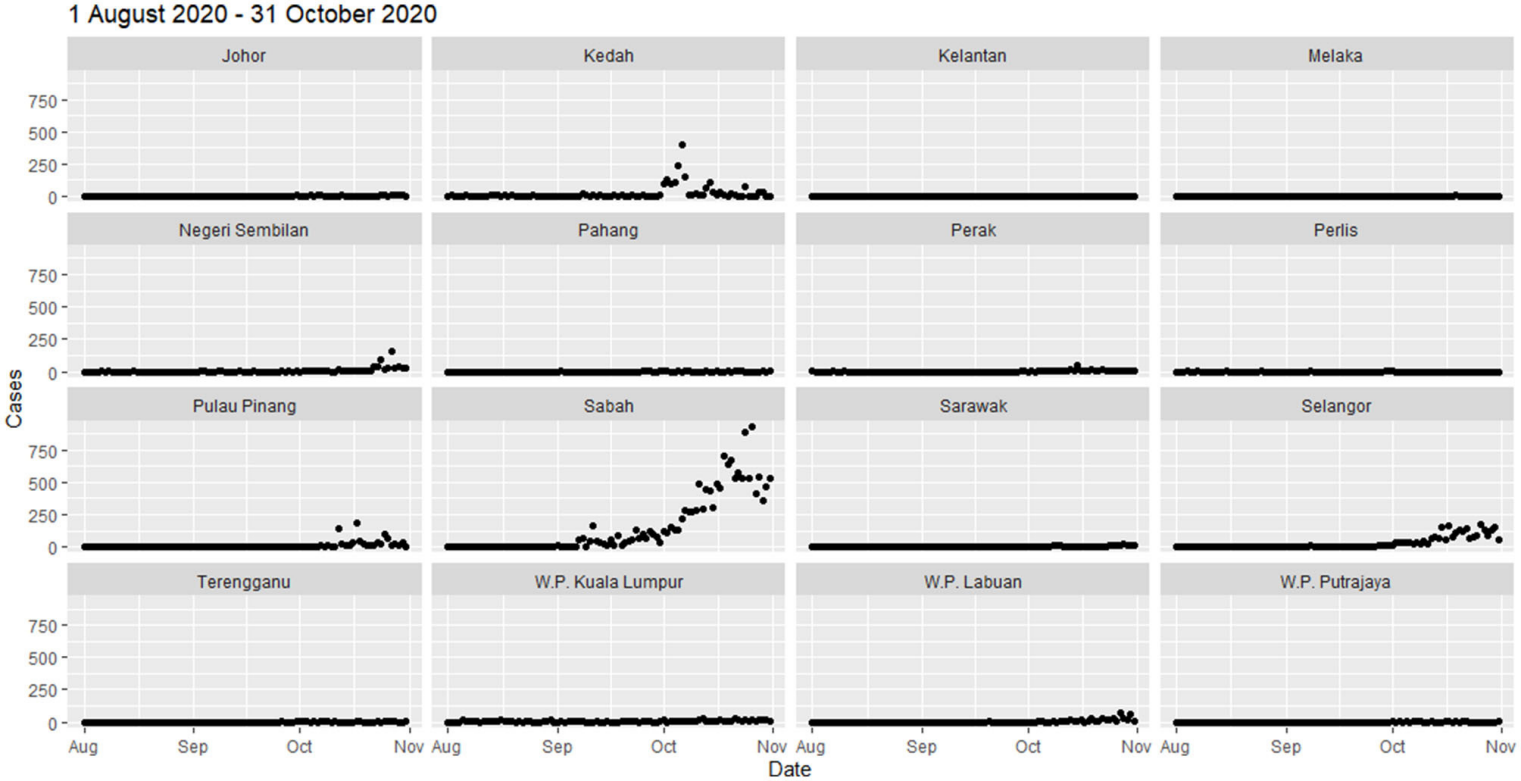
Total COVID-19 new cases by state from August 1 to October 31, 2020.

**Figure 5:**
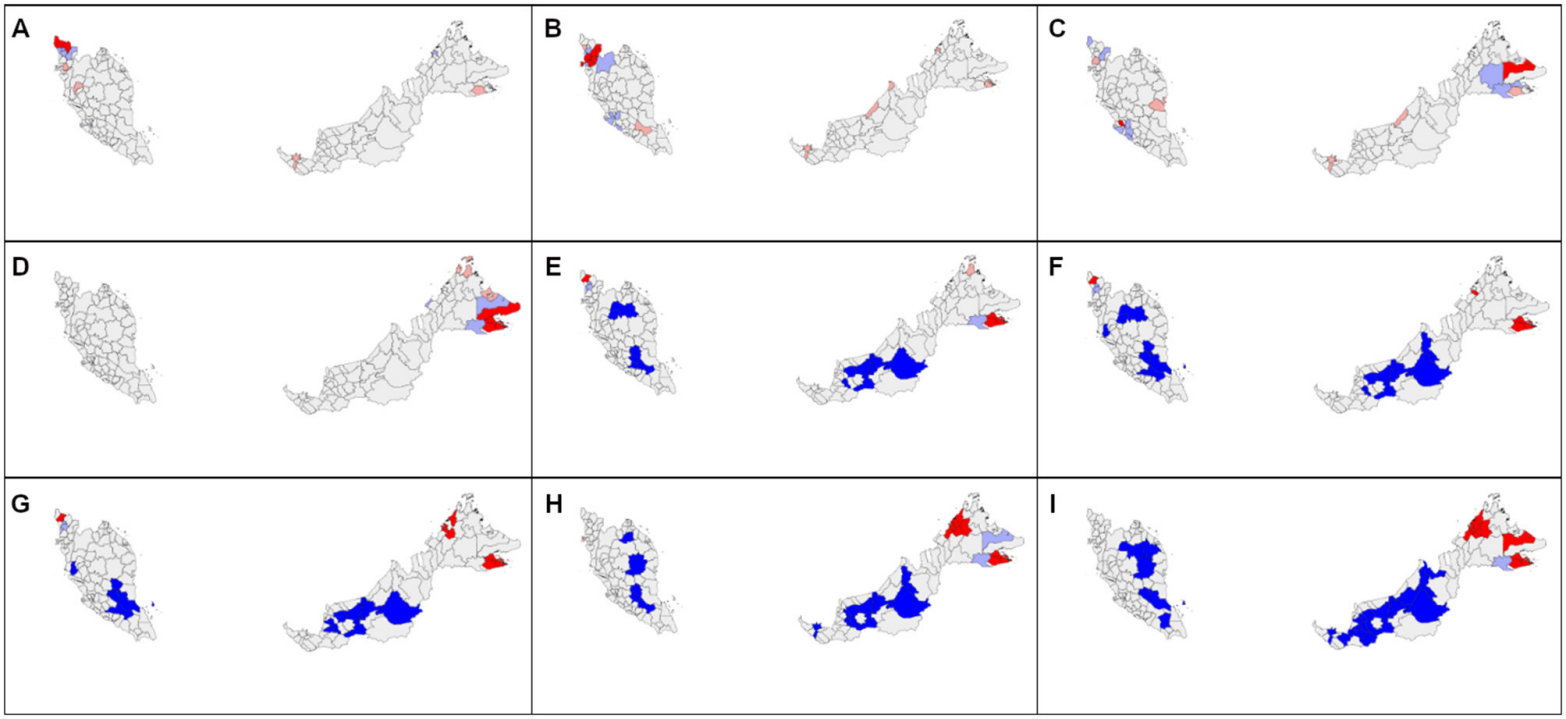
Spatial autocorrelation distribution of COVID-19 new cases by district in Malaysia at cumulative sum of current and 7 days retrospectively in 2020 on **(A)** August 5; **(B)** August 22; **(C)** September 1; **(D)** September 21; **(E)** October 5; **(F)** October 7; **(G)** October 8; **(H)** October 19; **(I)** October 22. Red color indicates high-high cluster, pink indicates high-low, blue indicates low-low, and light blue indicates low-high cluster.

From August to October 2020, clusters of COVID-19 cases were observed in the north of Peninsular Malaysia and in East Malaysia. Sabah experienced extremely high spikes in cases (total cases of 14,650) toward the end of October, whereas cases fluctuated in Kedah (2,015), Selangor (2,577), and Pulau Pinang (873) (Figure 4). A total number of cases in Selangor in August–October were three times higher than in May–July. In early August, the high-high cluster was concentrated in the northern Peninsular (Padang Terap, Kubang Pasu, Kedah; Perlis) (Figure 5). On August 22, 2020, the neighboring districts and states (Kuala Muda, Sik, Baling and Kulim, Kedah; Timur Laut, Barat Daya and Seberang Perai Utara, Pulau Pinang) became a high-high cluster. In the East Malaysia, high-high cluster was observed initially at east of Sabah (Kinabatangan) on September 1, 2020 and expanded to the neighboring districts (Lahad Datu, Semporna, Kunak, and Tawau) by September 21, 2020 (Figure 5). On October 7, 2020, the high-high clusters expanded from the east to the west (Penampang) of Sabah. After 12 days, the situation in Sabah worsens with the high-high clusters at eight districts in the west (Kota Kinabalu, Kota Belud, Tuaran, Tambunan, Papar, Ranau, Penampang, and Putatan) and three districts in the east (Semporna, Kunak, and Tawau). On October 22, 2020, one additional district (Kinabatangan, Sabah) was added to the high-high clusters (Moran's *I* = 0.57, *p* < 0.05). From November 1 till February 24, 2020, the high-high clusters scattered throughout Malaysia (Supplementary Figure S3). Selangor ranked highest in total cases (92,121 total new cases, 35.4%), followed by Sabah (37,325, 14%), Johor (33,864, 13%), and Kuala Lumpur (31,132, 12%) (Supplementary Figure S3). Selangor, Johor, and Kuala Lumpur showed an increasing trend, but in Sabah, total number of new cases gradually decreased. The Sabah's high-high cluster subsided in the east by November 13, 2020, but persisted in the west until January 29, 2021.

**Figure 6:**
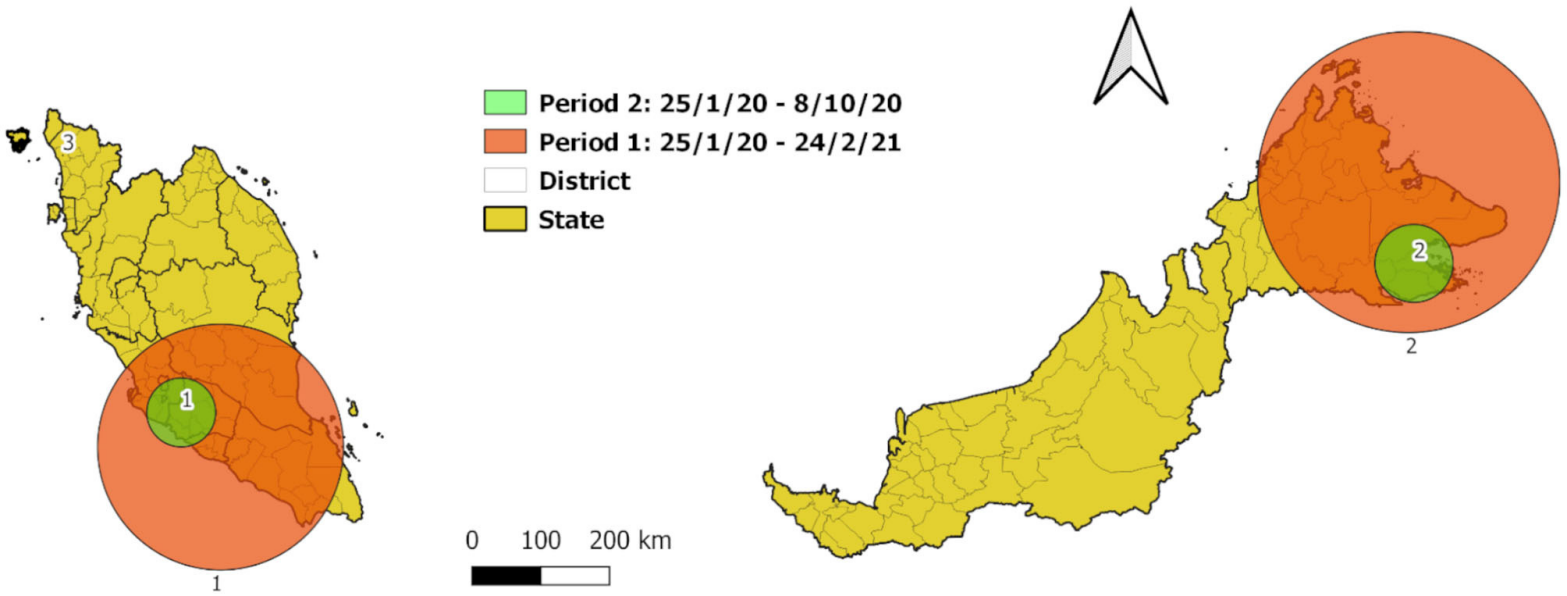
Spatial scan statistic of COVID-19 new cases by district in Malaysia at cumulative sum of current and 7 days retrospectively from January 25, 2020 to February 24, 2021.

Space-time scan was applied to two temporal period, (1) period of initial cases until introduction of vaccine and (2) period of initial cases until 1 day before initial cluster date in period 1. In the first period, a main cluster was detected with a radius of 178.8 km (RR = 11.93; *p* < 0.001; log-likelihood ratio 1344194.72), which spanned the following districts: Jasin, Melaka Tengah, Alor Gajah in Malacca, Tampin, Rembau, Kuala Pilah, Jempol, Port Dickson, Seremban, Jelebu in Negeri Sembilan, Muar, Batu Pahat, Kluang in Johor, Bera, Rompin in Pahang, Sepang, Kuala Langat in Selangor, Kuala Lumpur, and Putrajaya, from November 24, 2020 to February 24, 2021 (Figure 6, Table 1). A secondary cluster was also detected, comprised of 23 districts in Sabah with a radius of 218.77 km (RR = 5.31; *p* < 0.001; log-likelihood ratio 259019.17) from October 9, 2020 to February 8, 2021.

**Table 1:** Space-time clusters of COVID-19 cases from January 25, 2020 to February 24, 2021 at the district level.

**Cluster**	**Duration (days)**	**Observed**	**Expected**	**RR**	**LLR**	**# Of districts**	**Population at risk**
**Period 1: January 25, 2020–February 24, 2021**							
1^*^	2020/11/24–2021/2/24	1156183	210343.49	11.93	1344194.72	36	15,487,800
2^*^	2020/10/9–2021/2/8	324500	70564.82	5.31	259019.17	23	3,928,500
**Period 2: January 25, 2020–October 8, 2020**							
1^*^	2020/3/16–2020/6/10	29302	4404.23	9.37	34647.83	10	4,896,200
2^*^	2020/9/8–2020/10/8	12108	333.79	41.73	32512.96	4	1,041,400
3^*^	2020/10/2–2020/10/8	4930	30.64	170.11	20283.69	1	423,400

*RR, relative risk; LLR, log-likelihood ratio*.

* *p < 0.05*.

Three clusters were identified by space-time scan in the period 2 (Figure 6, Table 1). The main cluster was 50.12 km radius (RR = 9.37; *p* < 0.001; log-likelihood ratio 34647.832418), which encompasses the districts of Seremban, Port Dickson, Rembau, Jelebu, Kuala Pilah in Negeri Sembilan, Putrajaya, Sepang, Hulu Langat, Kuala Langat in Selangor, and Kuala Lumpur from March 16, 2020 to June 10, 2020 for more than 3 months, the population at risk of which is 4.7 times of population in the secondary cluster. The secondary cluster was 56.74 km radius in Kunak, Tawau, Semporna, Lahad Datu in Sabah (RR = 41.73; *p* < 0.001; log-likelihood ratio 32512.96) from September 8, 2020 to October 8, 2020 within 1 month. The third cluster was concentrated in Kota Setar, Kedah from October 2 to 8, 2020 (RR = 170.11; *p* < 0.001; log-likelihood ratio 20283.69).

## Discussion

In this study, we used R script to obtain the spatial autocorrelation of 374 time points (January 25, 2020–February 24, 2021) for 155 districts and three federal territories in Malaysia. Significant global Moran's I indices above 0.5 were observed in the initial periods of the second and third waves implying an impending outbreak. The daily local indicators of spatial autocorrelation depicted the disease spread dynamics in Malaysia across districts. Space-time clusters obtained from space-time statistics confirmed the high-risk areas identified using local Moran's I. Both daily Moran's I and number of high-high district clusters could be used as additional indices for monitoring COVID-19 spatiotemporal transmission intensity. A higher Moran's I value indicates that cases are clustered in an area that may potentially result in an outbreak to the residents in and surrounding the area.

Global Moran's I value above 0.5 (*p* < 0.05) was observed in the initial period of the outbreak (Figure 1B). Moran's I value first peaked above 0.5 during March 7 and 11, 2020 preceding a surge of COVID-19 cases in Selangor, Kuala Lumpur, and Putrajaya. This surge was due to a mass religious convention held in Kuala Lumpur from February 27 to March 3, 2020 (14), which resulted in the second wave of cases from February 26, 2020 to June 30, 2020 (23) in Malaysia. The second and third peaks of Moran's I values were in mid-September and end of October to early November, 2020, which also preceding a surge of COVID-19 cases of the third wave of COVID-19 in Malaysia. These values informed the spatial dynamics of the initial outbreak, in addition to the incidence rate and time-varying reproduction number (25).

The daily local spatial autocorrelation (LISA/local Moran's I) was able to measure the dynamics and intensity of the spatial spread of disease based on population at risk. Similar measures of spatiotemporal spread have been assessed in other countries, such as China (16), Italy (34), and Russia (35). In Malaysia, initially from January 25 to April 30, the spread of COVID-19 high-high cluster of new cases started in Selangor and Kuala Lumpur in the mid-west peninsular Malaysia on to the south and then west of Sarawak. The high-high cluster in Selangor receded after July 2020. From May 1 to July 31, high-high clusters were mainly reported in Selangor, Kuala Lumpur, Negeri Sembilan, Johor, and Sarawak. In early August, the spatial autocorrelation high-high cluster initiated in Kubang Pasu, Kedah was traced to an index case with the super spreader strain D614G, that spread to neighboring Perlis and Pulau Pinang state called the Sivagangga cluster (36). The high-high cluster in Sabah can be traced to an outbreak in a police detention center in Lahad Datu, Sabah known as the Benteng Lahad Datu cluster with total contact cases of 1,146 (11), which affects neighboring districts that include Tawau, Sandakan, Kinabatangan, and Tuaran. Since September 14, 2020, the high-high cluster in Sabah had expanded from two districts to five in 8 days. After the Sabah state elections were held on September 26, 2020, cases surged exponentially and the high-high cluster increased to 12 districts, from the east coast to the western part of Sabah. This may be attributed directly (70% of cases within Sabah) or are spillover effects (64.4% of cases in the rest of Malaysia) of the Sabah election (37). The total number of cases in the period of August to October 2020 was 7.6 times the figure 3 months before. From November 2020 to 24, February 2021, the high-high clusters shifted from Sabah to Selangor and Negeri Sembilan, then to Pahang, Johor, and Sarawak. Phylogenetic analysis of new B.1.524(G) lineages support claims that the cases in Selangor came from Sabah (38).

Spatiotemporal clustering obtained using space-time scan statistics provided additional information on the dispersal of the disease. The cluster with the highest relative risk was RR = 11.93, in the southwest of peninsular Malaysia, that is, the districts in the cluster had 11.93 times the risk of COVID-19 compared to districts outside the cluster in the same period, with 15,487,800 population at risk (nearly half of the population in Malaysia). Space-time scan statistical analysis has been widely utilized to study other diseases such as dengue, zika, MERS (39–41). Previous studies using space-time scan for COVID-19 discovered that Hubei province has higher risk than the other region in China (42) whereas a Korean study found higher risk in Daegu City than neighboring province (43). A retrospective spatial scan study in Southeast Asia during January 13, 2020 and March 16, 2020 further confirmed that Malaysia and Singapore were the most likely cluster between March 4–March 16, 2020 (RR = 72.07, LLR = 1910.08, *p* < 0.001) (44). The most likely cluster for the period 2 (January 25 to October 8, 2020) consisted of Negeri Sembilan, Putrajaya, Kuala Lumpur, and Selangor (10 districts) in this study correlates with a state-level spatial scan analysis study in Malaysia (45).

Identifying the areas with high spatial clustering of cases assists in public health control measures. The spatial autocorrelation analysis showed that COVID-19 is highly likely to have similar high number of cases at the adjacent districts. Significant spatial association of COVID-19 cases was also discovered in neighboring region in China (46). It is indisputable that cross-boundary transmission may occur across administrative boundaries in short period. The analysis could be used as a tool for decisions on shrinking and expanding movement control boundaries. Space-time scan statistics analysis exhibited the connection of daily clusters in space and time with a flexible window setting, but is restricted to the shape of the scanning window and might include neighboring low case districts that fall in the buffer area. Whereas, local Moran's I is able to delineate the neighboring cluster and is not limited to the shape of a scanning window. Whereas, in this study, we focused on daily local Moran's I, Hohl et al. (47) were able to detect emerging clusters using daily prospective space-time scan statistic. Both spatial autocorrelation and scan statistics could be taken as reference as they complement each other (48, 49). Analysis at smaller units of aggregation, such as sub-district or residential area, will better reflect the real-time local clustering events, and thus, smaller area public health data need to be made available in setting geographical policy. Therefore, understanding the spatiotemporal clustering situation could provide valuable information in measuring the disease dispersal pattern and intensity of an epidemic.

Our study has several limitations. First, for Moran's I and LISA analysis, the irregular shapes of the districts affected the weight matrix of the spatial autocorrelation. This issue can only be resolved using regular spatial grids and aggregation of the address or point-level data. Second, we could not perform spatial autocorrelation analysis on the number of deaths by districts as the open-source data on COVID-19 deaths were limited only to state level. Without these data, the interaction and dynamics of the cases and deaths could not be elucidated from the spatiotemporal perspective. Third, there may be underreporting of cases; nevertheless, the clustering effect revealed the underlying risk of the transmission. Fourth, there is the well-known modifiable areal unit problem (MAUP) which occurs when using aggregated data that thwarts obtaining consistent results with different spatial analysis levels. Future research is suggested to conduct multiscale and multizonal system analyses to address this problem (50).

## Conclusion

In conclusion, we were able to estimate the spatiotemporal trend of COVID-19 in Malaysia from January 25, 2020 to February 24, 2021 using Moran's I index and space-time scan statistics. Daily monitoring of these indicators could be useful additional information for public health managers to assess the spatiotemporal risk of the epidemic. This analysis can also be used in real-time monitoring of the outbreak. For epidemic spread and density forecasting, both analyses need to be in smaller spatial and time units to enable prospective estimation of potential outbreaks. Future studies are needed to study the spatiotemporal variation of Rt values in determining the correlation of transmission and preventive measures and to compare the cluster patterns obtained in this study with those using advanced Bayesian spatiotemporal models and machine learning spatial modeling.

## Data Availability Statement

Publicly available datasets were analyzed in this study. This data can be found at: https://github.com/MoH-Malaysia/covid19-public.

## Author Contributions

YC, KL, SM, MC, and NM contributed tremendously to the production of this manuscript. QR managed and cleaned the data. CK, FL, and BG reviewed and gave technical advisory to the manuscript and contributed essential revisions. All authors have read and approved the manuscript.

## Funding

This work was accomplished under the project of National Institute of Health, Ministry of Health Malaysia (NMRR-21-367-58473).

## Conflict of Interest

The authors declare that the research was conducted in the absence of any commercial or financial relationships that could be construed as a potential conflict of interest.

## Publisher's Note

All claims expressed in this article are solely those of the authors and do not necessarily represent those of their affiliated organizations, or those of the publisher, the editors and the reviewers. Any product that may be evaluated in this article, or claim that may be made by its manufacturer, is not guaranteed or endorsed by the publisher.
