# Supplementary material for: Assessing the Spatiotemporal Spread Pattern of the COVID-19 Pandemic in Malaysia
Source: Front Public Health. 2022 Mar 4;10:836358. doi: 10.3389/fpubh.2022.836358 (PMC8931737; doi:10.3389/fpubh.2022.836358)
Supplement: Supplementary file 1 [file Table_1.docx]

Supplementary Material


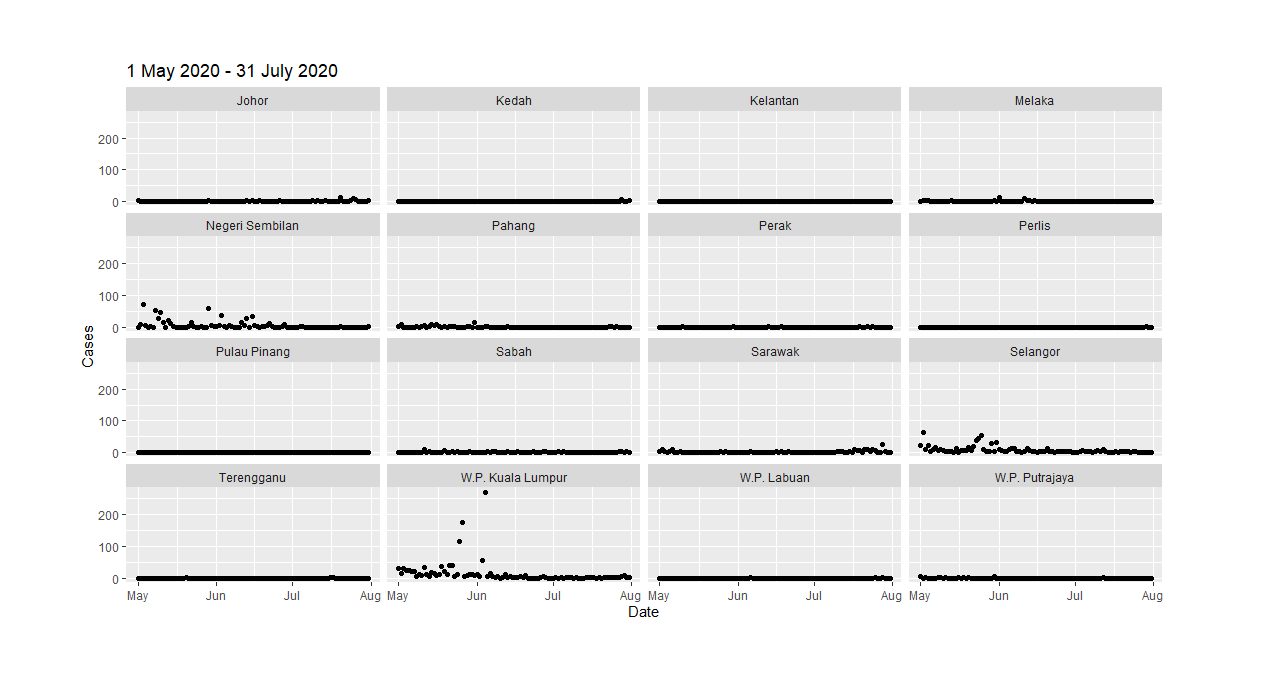


**Supplementary Figure 1.** Total COVID-19 new cases by state from May 1 to July 31, 2020.


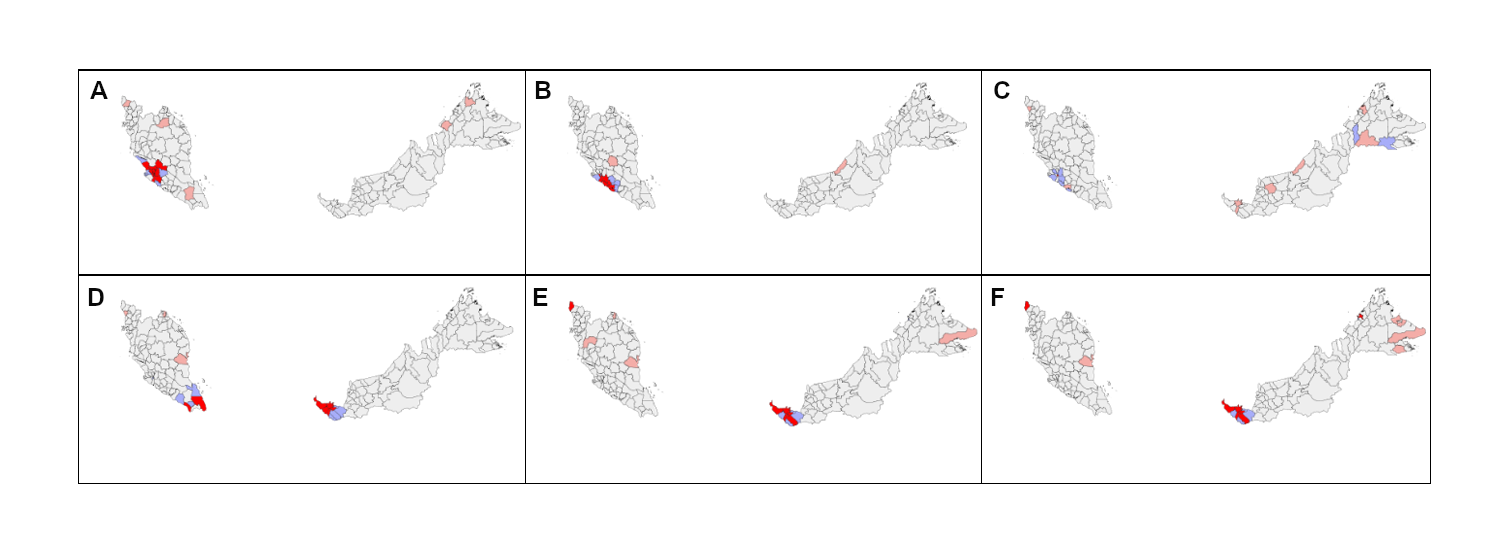


**Supplementary Figure 2.** Spatial autocorrelation distribution of COVID-19 new cases by district in Malaysia at cumulative sum of current and 7-days retrospective in 2020 on **(A)** May 23; **(B)** June 16; **(C)** July 14; **(D)** July 26; **(E)** July 29; **(F)** July 31. Red color indicates high-high cluster, pink indicates high-low, blue indicates low-low, and light blue indicates low-high cluster.


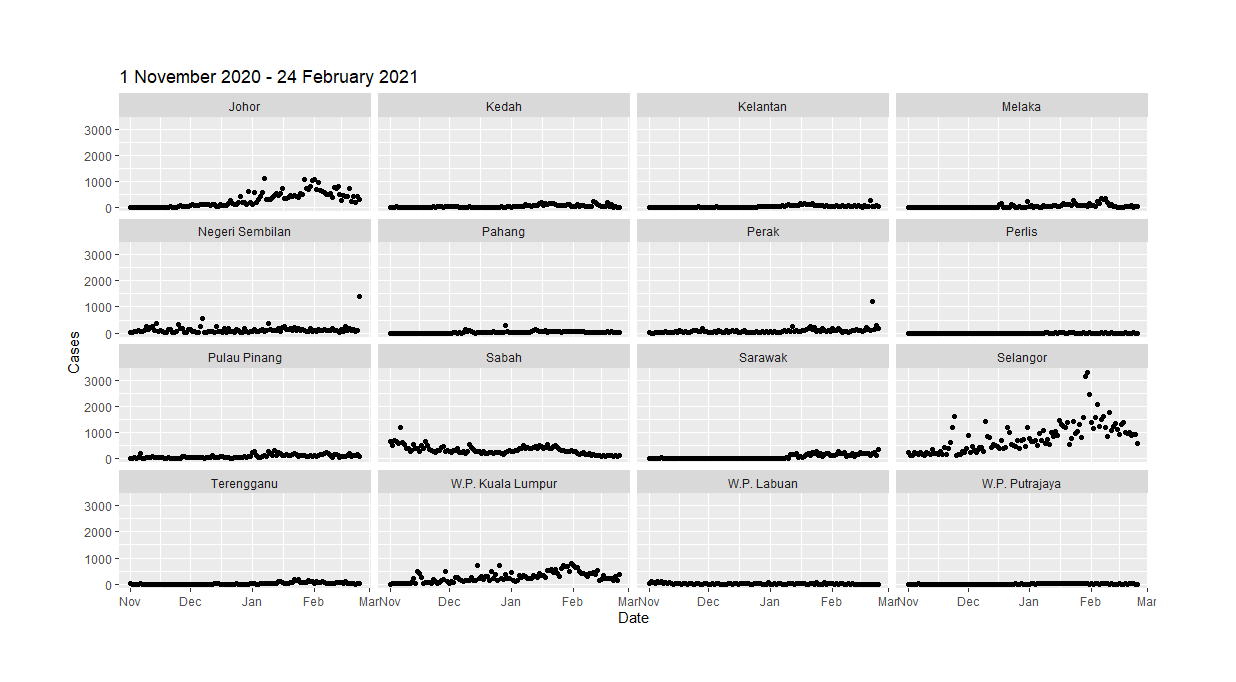


**Supplementary Figure 3.** Total COVID-19 new cases by state from November 1, 2020 to February 24, 2021.


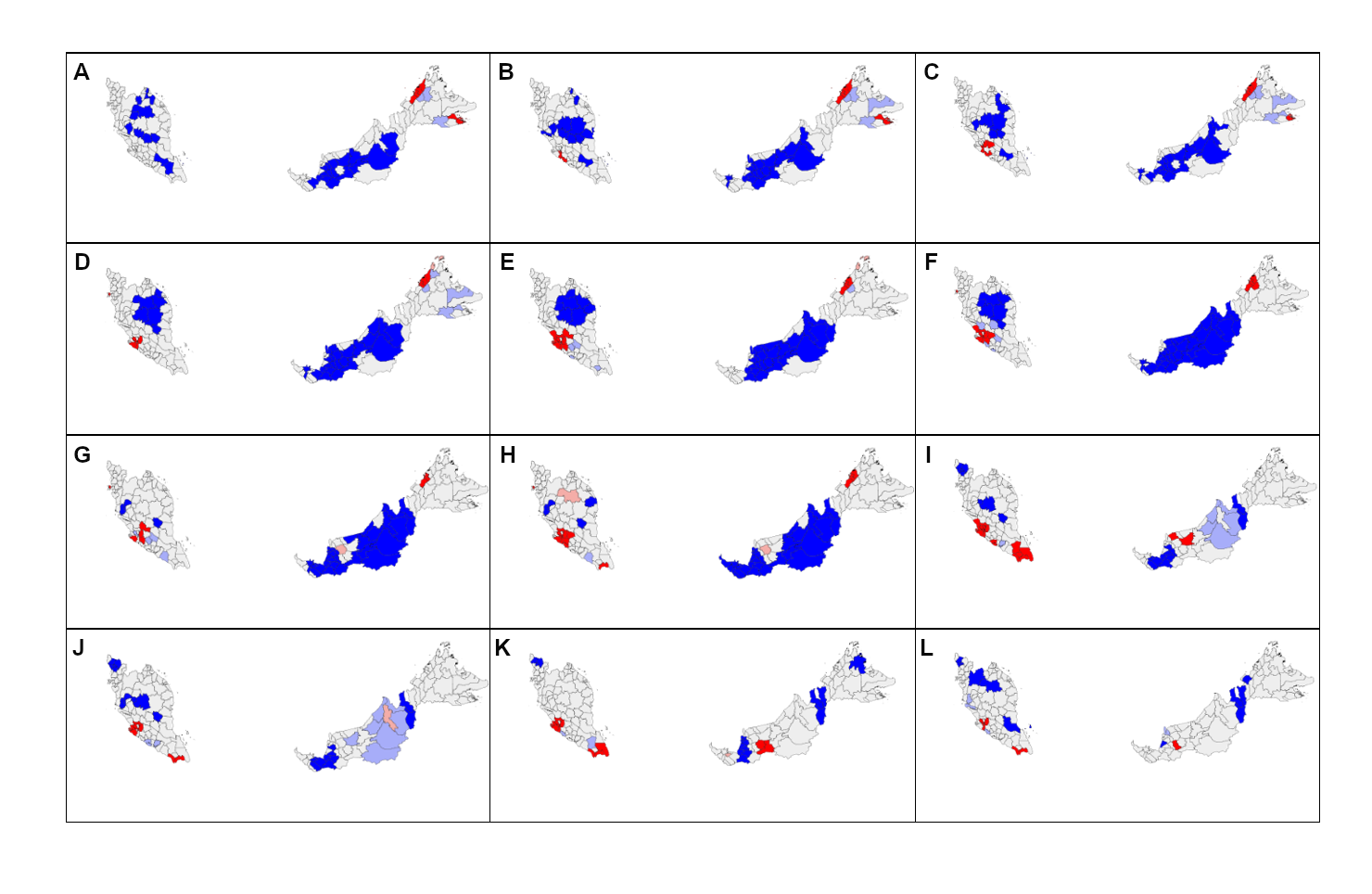


**Supplementary Figure 4.** Spatial autocorrelation distribution of COVID-19 new cases by district in Malaysia at cumulative sum of current and 7-days retrospective on **(A)** November 3, 2020; **(B)** November 11, 2020; **(C)** November 13, 2020; **(D)** December 2, 2020; **(E)** December 28, 2020; **(F)** January 2, 2021; **(G)** January 12, 2021; **(H)** January 17, 2021; **(I)** February 6, 2021; **(J)** February 7, 2021; **(K)** February 18, 2021; **(L)** February 24, 2021. Red color indicates high-high cluster, pink indicates high-low, blue indicates low-low, and light blue indicates low-high cluster.
